# PairWise Neighbours database: overlaps and spacers among prokaryote genomes

**DOI:** 10.1186/1471-2164-10-281

**Published:** 2009-06-25

**Authors:** Albert Pallejà, Tomàs Reverter, Santiago Garcia-Vallvé, Antoni Romeu

**Affiliations:** 1 Department of Biochemistry and Biotechnology, Rovira i Virgili University, Tarragona, Catalunya, Spain

## Abstract

**Background:**

Although prokaryotes live in a variety of habitats and possess different metabolic and genomic complexity, they have several genomic architectural features in common. The overlapping genes are a common feature of the prokaryote genomes. The overlapping lengths tend to be short because as the overlaps become longer they have more risk of deleterious mutations. The spacers between genes tend to be short too because of the tendency to reduce the non coding DNA among prokaryotes. However they must be long enough to maintain essential regulatory signals such as the Shine-Dalgarno (SD) sequence, which is responsible of an efficient translation.

**Description:**

PairWise Neighbours is an interactive and intuitive database used for retrieving information about the spacers and overlapping genes among bacterial and archaeal genomes. It contains 1,956,294 gene pairs from 678 fully sequenced prokaryote genomes and is freely available at the URL . This database provides information about the overlaps and their conservation across species. Furthermore, it allows the wide analysis of the intergenic regions providing useful information such as the location and strength of the SD sequence.

**Conclusion:**

There are experiments and bioinformatic analysis that rely on correct annotations of the initiation site. Therefore, a database that studies the overlaps and spacers among prokaryotes appears to be desirable. PairWise Neighbours database permits the reliability analysis of the overlapping structures and the study of the SD presence and location among the adjacent genes, which may help to check the annotation of the initiation sites.

## Background

The availability of fully sequenced genomes has grown exponentially over the past few years. There is a huge variety of environments for the prokaryote species, as well as different metabolic and genomic complexities. However, prokaryote genomes have common architectural principles [1]. The prokaryote genomes contain protein-coding genes, structural RNAs and spacers between genes which are thought to typically contain regulatory signals [2] and the origin of replication sequence [3]. These spacers tend to be short because of the selective pressure to minimize the non-functional DNA in prokaryotes [2,4]. It is a consistent feature of these genomes that the genes often overlap their coding sequences [5]. Under this scenario of genomic compactness due to their physically small environments, the overlapping genes follow the rules that impose the structure of the genetic code and the spacers between genes must adapt their lengths to the requirements of the regulatory signals [2].

One of the regulatory signals that we can find between genes is the Shine-Dalgarno (SD) sequence [6]. The SD sequence is a motif, 5'-GGAGG-3', located at the 5' of the initiation codons and is complementary to the sequence, 5'-CCUCC-3', located at the end of the 16S rRNAs [6]. The ribosome does not need a perfect distance between the SD sequence and the start codon for the initiation of translation. However, it has been studied that when the SD resides within the 4 nucleotides from the initiation codon or when it is located as far as 13 nucleotides from the initiation codon, gene expression is decreased drastically [7-9]. The prokaryote species seem to have preferred distances between the SD and the start codon and these distances vary among the species [10], although this sequence has been found mostly from the 7th to the 12th base upstream from the start codon [10-12]. The location of the SD can help to correct the gene annotations [13] and could influence the spacing length and the stop codon usage [14].

Among the prokaryote genomes there is a huge amount of examples of overlapping genes [15-19]. The overlapping lengths tend to be short because of the selective pressure against long overlaps, as the existence of long overlapping reading frames increases the risk of deleterious mutations. The co-directional overlaps are the most common overlaps, which reflect that this is the most common orientation for a gene pair due to the tendency to be grouped in operons in prokaryote genomes [20-22]. Among the co-directional overlaps the 4 bps overlap is extremely common [5,15,23,24], which permits the upstream stop codon and the downstream start codon overlap and the gene pair is thought to be translationally coupled [25]. The co-directional and divergent overlapping genes can arise by 5'-end elongations when the downstream gene adopts a new start codon within the upstream coding sequence [23], while the co-directional and the convergent overlapping genes can arise by 3'-end extensions after a loss codon event [16]. Overlaps in prokaryotes have been hypothesized to be involved in reducing the genome size in order to increase the density of genetic information [17,24,26-28], and in regulating gene expression through translational coupling of functionally related polypeptides [5,24,26,29,30]. In addition, other authors have used the overlapping pairs as genetic markers for phylogenetic inferences due to its high conservation [31,32]. Overlapping genes are better conserved across the species than non-overlapping genes [19]. The extent of conservation of the overlapping pairs correlates with the evolutionary distances between the pairs of species [15].

The overlapping genes, as a common structure of the prokaryote genomes, and the spacers between genes are structural features worth studying in prokaryotes. However, the analysis of both the overlapping genes and the spacers between genes is often affected by genome annotation errors [33-35]. An accurate annotation would facilitate the experiments as well as the bioinformatic analysis of gene regulation and gene structure [36]. In this interactive database is stored all the overlapping genes and the spacers of 678 fully sequenced prokaryote genomes. The aim of this database is to provide the users with useful information about the overlapping genes and the spacing lengths between adjacent genes. The conservation of the overlaps across the species and the SD presence and location within the intergenic regions or the overlapping sequences can be analysed. Obviously, the quality of the information given depends on the quality of the genome annotations. In fact, this database can be used to analyse suspicious cases of genome annotation errors such as wrong initiation sites or false gene predictions.

## Construction and content

### Retrieval of the Spacing lengths and the Overlapping genes

The complete genome sequences of 678 prokaryote genomes were downloaded from the NCBI ftp site . Scripts implemented in Perl language were performed to extract and analyse the spacers and the overlaps between adjacent genes and all the information related (spacing & overlapping lengths, spacing & overlapping sequences, gene orientations, phases, protein functions, gene COGS and stop & start codons of the genes). The internal gene ids in this database have been formed by joining the GenBank Accession Number with the gene name. For instance, the gene id for the HI0038 gene from *Haemophilus influenzae *Rd KW20 is NC_000907.HI0038. Furthermore, each overlap and spacer between adjacent genes has an internal id. The spacing lengths and the overlapping genes have been classified into three types according to their transcriptional direction [2,16,26]: i) unidirectional (genes in the same strand overlapping the 3'-end of an upstream gene and the 5'-end of a downstream gene), ii) convergent (genes in opposite strand overlapping the 3'-ends) and iii) divergent (genes in opposite strand overlapping the 5'-ends). In this database we use the term co-directional instead of the unidirectional term. In order to study the phases between adjacent genes, as other authors have previously done [5,19,23], we defined three overlapping phases: (i) phase 0 where the downstream gene is in frame with the upstream gene (lengths n = ..., -12, -9, -6, -3, 0, 3, 6, 9, 12, ...), (ii) phase 1 where the downstream gene is in the reading frame +1 relative to the upstream gene frame (lengths n = ..., -11, -8, -5, -2, 1, 4, 7, 10, ...) and (iii) phase 2 where the downstream gene is in the reading frame +2 relative to the upstream gene frame (lengths n = ..., -10, -7, -4, -1, 2, 5, 8, 11, ...).

### Location of the SD sequence and determination of its binding strength

We extracted the 16S rRNAs from the NCBI ftp site . For each 16S rRNA sequence of each organism we looked at the 5' direction for the first instance of the three letter motif, 5'-GAU-3', which was found consistently on the 5' end tails of the 16S rRNAs with known structures. The location of this motif was used to define, up to the end of the 3' tail, the 16S rRNA tail of each organism. For species that have two or more copies of the 16S rRNA gene, we calculated the consensus sequence of all the tails. If the different tails observed did not follow a consensus, then we used the majority of the 16S rRNA gene tails. All the 16S rRNA tails of the 678 organisms were examined manually. The SD sequences for 678 prokaryote genomes have been predicted using computer calculations of the base pairing free energy between translation initiation regions and the 16S rRNA 3' tail. The method used was developed by Starmer and co-workers [12]; and the scripts to calculate the free energies were downloaded from  and were included in our Perl scripts. We located the SD sequence by the position of the lowest ΔG° value calculated from 35 bps upstream to the initiation codon to 35 bps downstream from the initiation codon. The gene was assumed not to have the SD sequence if ΔG° > -3.4535 Kcal/mol and to have SD sequence if ΔG° ≤ -3.4535 Kcal/mol. The threshold used is based on the work of Ma and co-workers [10]. The gene was assumed to have a strong SD sequence if ΔG° ≤ -8.4 Kcal/mol, which is the value obtained from the optimal base pairing between the 16S rRNA and the original SD sequence 5'-GGAGGU-3' [13]. In order to point the exact SD position we used the relative spacing parameter [13], that means that we calculated the distance between the first residue of the start codon and the 5' A of the rRNA sequence 5'-ACCUCC-3' in each position around the start codon. If the SD motif is located before the start codon the relative spacing will be negative, while if the SD motif is located after the start codon the relative spacing will be given as a positive number. Regardless the gene pair orientation, the SD information and the graph of the ΔG° values is given for the upstream and the downstream gene.

### Database Construction

**Figure 1:**
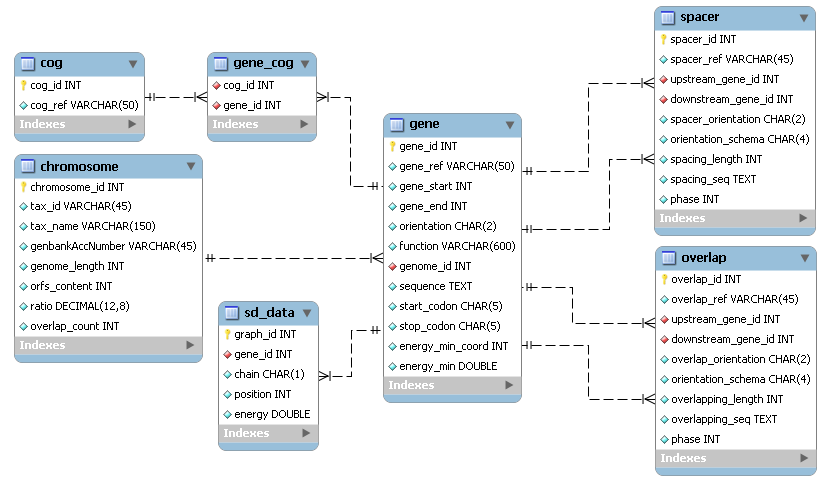
**Entity-relationship model of the MySQL database**. Schema of the data model designed and translated to a MySQL database.

The huge amount of data generated required a data model to make it possible to work with this data efficiently. The Entity-relationship model, showed in Figure 1, was designed and transformed in a MySQL database. A web application was developed using the framework web TurboGears. This Python framework MVC (Model-View-Controller) is an advanced tool to create data consulting systems quickly, efficiently and consistently. The BLAST search tool [37] was installed in our server and is used to study the conservation of the gene overlaps. All the graphs are generated at the user side by a Java Script library named PlotKit.

## Utility

We have developed an interactive and intuitive database that currently contains 1,956,294 gene pairs from 678 fully sequenced microbial genomes. The database is freely available at the URL . Basically, this database provides information about the overlapping genes and the spacers between genes among the prokaryote genomes. Users can access to the information through three browsers and an advanced search engine, which are described below. In addition, this database offers the possibility of downloading the raw data and a Database Schema (Figure 1) in the Downloads section. They can find information about the overlaps and the spacers with the species name or the GenBank Accession Number, with the gene id (they can use the gene name, the short gene name or the PID) or with the internal gene id (described above in the Construction and Content section). While the users are typing the species name or any gene id the search engine helps to complete the name or the id. Interestingly, by clicking on the "TagClouds", the user can get a list of the species contained in the database, which can be sorted by the number of overlaps in a genome or by genome length in order to check at a glance the genomes with more overlaps or longer genomes. Furthermore, the database is able to provide the users with reports in TSV format at every step of their consultation just on clicking the Download TSV Data buttons.

### The Genome browse

With this browser, users can find general information about the genomes and connect to the overlapping genes or the spacers between genes contained in the genome. They can access this information by typing the name of the species (by tax name) or the GenBank Accession Number (by genbank). If they do not remember the species name or the GenBank Accession Number by clicking on "Genome (List)" the users can consult an exhaustive list of the species contained in this database and their GenBank Accession Numbers. Once the user has made a genome search, the first page obtained gives basic features of the genome including the Species name, the GenBank Accession Number, the TaxID, the genome length, the number of ORFs in the chromosome, the number of overlaps and spacers in the genome, the overlaps between ORFs ratio in the chromosome and the number of co-directional, convergent and divergent overlaps tabulated and represented graphically. By clicking the number of overlaps a list of the overlaps contained in the genome is displayed on a new page, while on clicking the number of spacers a list of the spacers contained in the genome is displayed on another new page.

### The overlapping genes browse

The users can analyse the overlapping genes in a genome or a particular overlap of interest (by gene or by internal id). Once the user has made a genome search, the first page obtained has a list of the overlaps with the overlapping genes and their orientations as well as the distribution of the overlapping lengths represented graphically. The representation of the overlapping length distribution gives a general idea about the most common overlaps and the most common overlapping phases in the genome. Each overlap id leads to a detailed new page of the overlap including five labels that provide: overlap information, upstream gene information, upstream gene sequence, downstream gene information and downstream gene sequence. The overlap information label (*General Info *label) provides the internal id, chromosome name, the orientation, the overlapping phase, the overlapping length and the overlapping sequence. The upstream and downstream gene information labels (*Upstream Gene *and *Downstream Gene *label respectively) show the gene name, the gene function, the gene COG, the stop codon and the start codon. Also, on these labels is given information related to the SD location (position of the minimal ΔG° value and minimal ΔG° value) and the ΔG° values in the translation initiation region is represented graphically. The SD related information will be given in the upstream or in the downstream label depending on the gene pair orientation. The labels *Fasta Up *and *Fasta Down *contain the upstream and the downstream gene sequence in fasta format. Above the sequences there is a BLAST button. By clicking on it, the gene sequence is directly pasted in the BLAST local search engine and the conservation of one overlap across the species can be analysed. Interestingly, in the PairWise Neighbours database, the user can define the Expected threshold of the BLAST search engine among other features. Therefore the user can decide the threshold used to study the similarity among orthologous genes in order to analyse the overlapping pair conservation. In the BLAST results, by clicking on any hit, the information of the overlap is displayed on a new page.

### The spacers browse

The users can analyse the spacers between adjacent genes in a genome or a particular spacer of interest (by gene or by internal id). If the user makes a genome search, a bar chart of the spacing lengths of the genome is shown and the user can have a first view of the most common spacers in the genome. Below a list of all the spacers in the genome is displayed, providing the internal id, the genes separated by the spacer and their orientation. By clicking any internal id all the information about the spacer is displayed on a new page. On this page there are three labels that give information about: the spacer, the upstream gene and the downstream gene. Basically the information given in the fields on a general information label (*General Info *label) is the same as the fields on a *General Info *label of an overlap. However, the user can find the Spacing length instead of the Overlapping length and Spacer sequence instead of Overlapping sequence. The information provided on the *Upstream *and *Downstream Gene *labels is the same as that on the overlap labels and the SD related information is also given depending on the gene pair orientation.

### Advanced Search engine

In this Advanced Search it is possible to study the functionality of the genes more widely. The user can make correlations between the COG classes and the gene orientations or between the COG classes and the overlapping or spacing lengths among the prokaryote genomes. Furthermore, the user can retrieve the gene set of each organism without SD sequence, with SD sequence and with a strong SD sequence by just selecting the organism and the corresponding energy threshold (the energy thresholds are explained above in the Construction and Content section).

## Discussion

In this Discussion section we give a few examples that we find interesting to illustrate the uses that can be attributed to the PairWise Neighbours database.

### Conservation of gene overlaps

**Figure 2:**
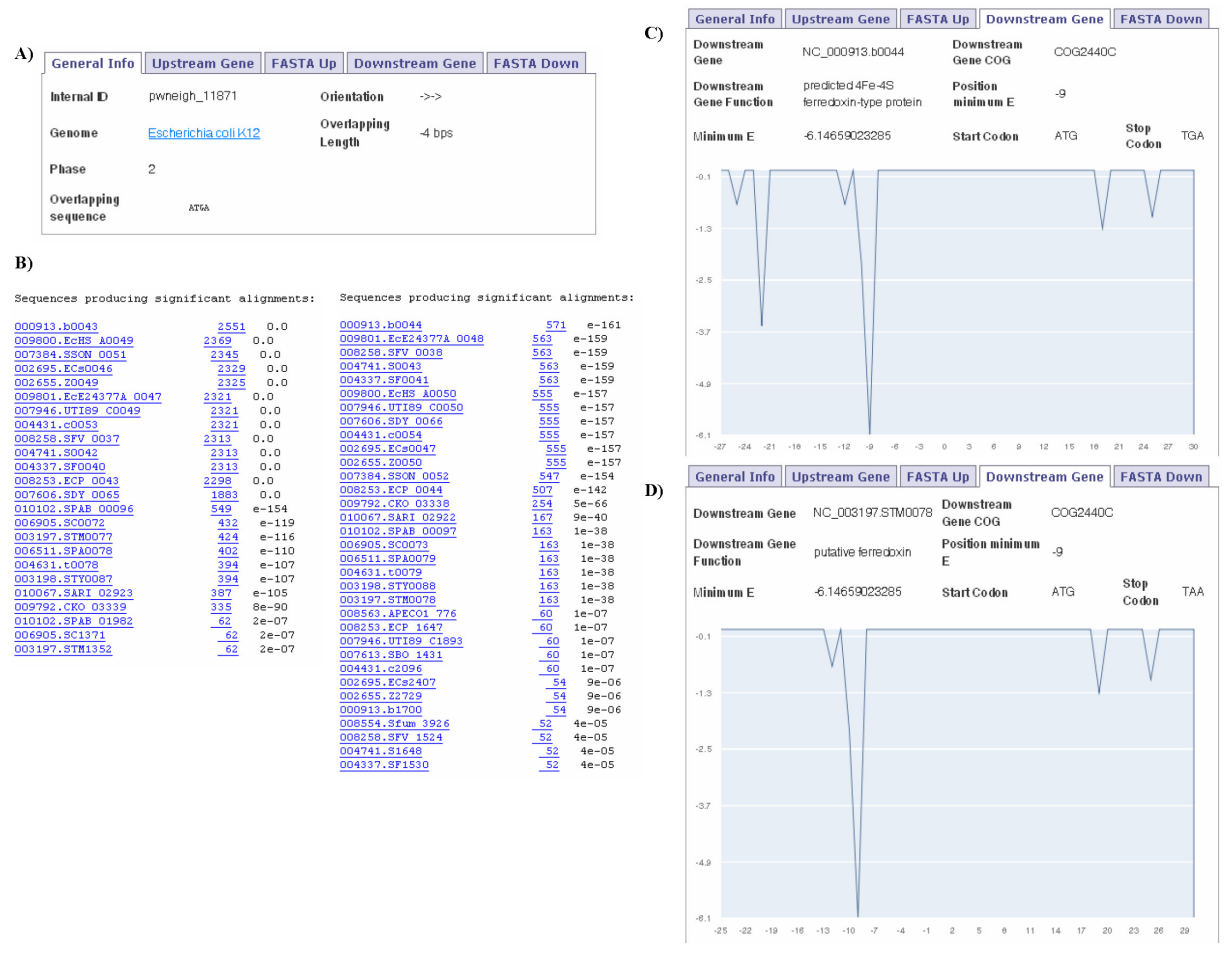
**Study of a 4 bps overlap conservation**. Compilation of images that the users can find when they are studying the conservation of an overlap. General Info label shows information about the 4 bps overlap between NC_000913.b0043 and NC_000913.b0044 genes (A). The BLAST results give an idea of the conservation of the overlap across the species (B). Information given on the NC_000913.b0044 *Downstream Gene *label provides gene details (gene function, gene COG, start and stop codon), SD related information (position of minimal ΔG° value, minimal ΔG° value) as well as a graph of the ΔG° values along translation initiation region (C). The same information is given for the NC_003197.STM078 gene (D), which is an orthologous gene of NC_000913.b0044.

The first one is about the gene couple NC_000913.b0043 and NC_000913.b0044 of *E. coli *K12, which code for two proteins 4Fe-4S ferredoxin-type and have the COG ids COG0644C and COG2440C respectively (Figure 2). These genes are overlapping 4 bps. From the upstream and downstream sequence labels it is easy to study the conservation of the gene pair, using the BLAST button. The BLAST results show 24 genes with high similarity (E Value < 2e^-7^) to the NC_000913.b0043 gene and 33 genes with high similarity (E Value < 4e^-5^) to the NC_000913.b0044 gene (Figure 2). By clicking on a gene id in the BLAST results, information about the overlap that involves the gene is displayed on a new page. Most of the genes similar to the NC_000913.b0043 gene have their adjacent gene in the group of similar genes to the NC_000913.b0044 gene and the majority of these gene pairs are overlapping 4 bps. Therefore it is a conserved overlap, particularly across the Enterobacteria species. Interestingly, we also find high conservation in the location of the SD sequence. Analysing the SD information for the NC_000913.b0044 gene (*Downstream Gene *label in Figure 2) we observe a drop in ΔG° value at 9 nucleotides to the start codon. This SD position is conserved among Enterobacteria species. Figure 2 shows the information for the NC_003197.STM0078 gene of *Salmonella typhimurium *LT2, which overlaps 4 bps with the NC_003197.STM0077 gene. These genes are similar to the *E. coli *K12 gene pair analysed. The NC_003197.STM0078 gene shows a drop in ΔG° value at 9 nucleotides to the start codon, as it happens in the NC_000913.b0044 gene of *E. coli *K12. This indicates that the SD sequence is located along the 3'-end of a previous coding sequence and it might suggest that the SD locations of conserved gene pairs can also be highly conserved.

**Figure 3:**
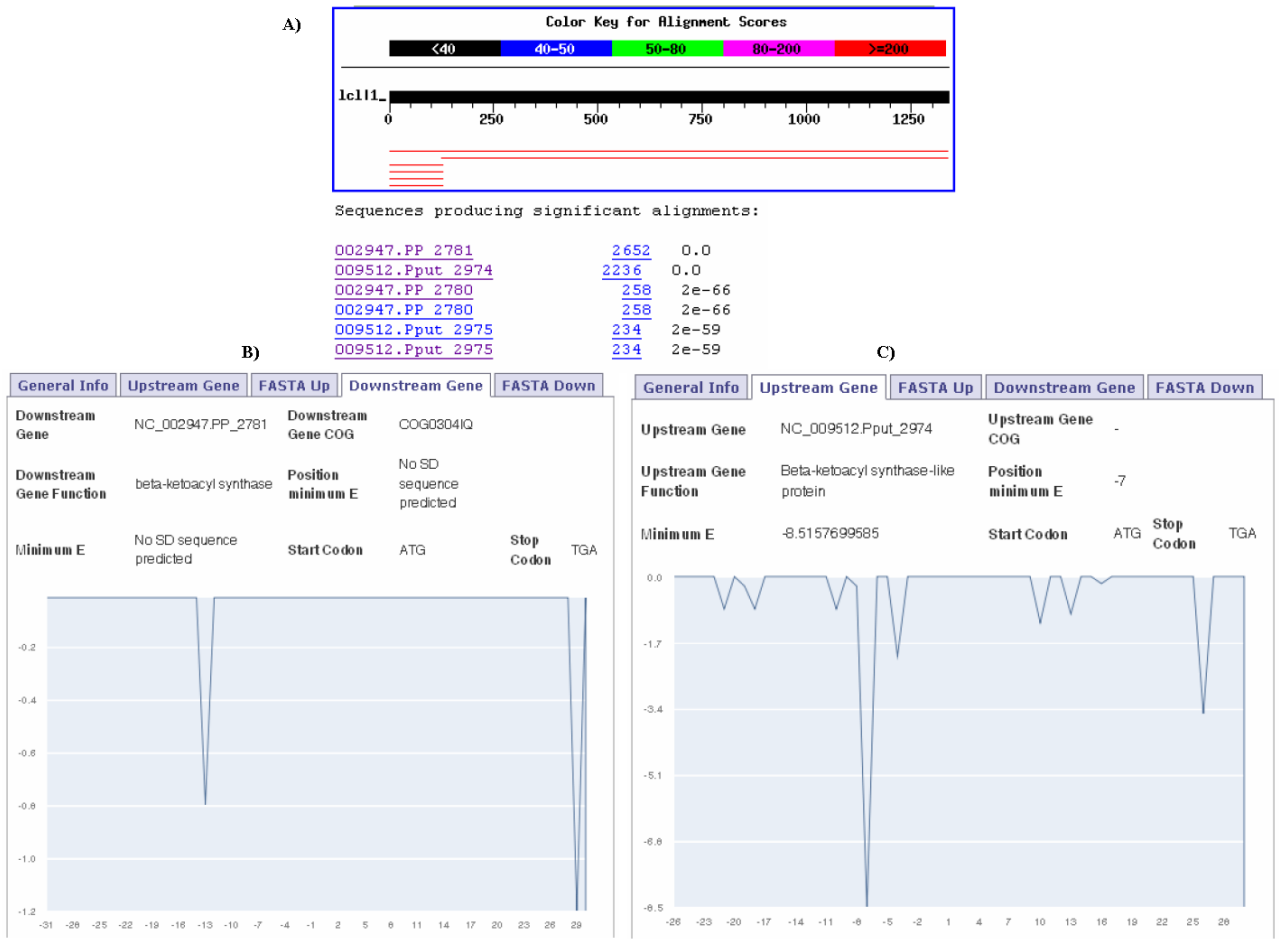
**Study of a 130 bps incorrectly annotated overlap**. The BLAST results show that the gene NC_002947.PP_2781 of *P. putida *KT2440 is longer than its orthologous gene NC_009512.Pput_2974 in *P. putida *F1 (A). This difference in length indicates that the 130 bps overlap between NC_002947.PP_2780 and NC_002947.PP_2781 is not conserved and thus not reliable. In the NC_002947.PP_2781 *Downstream Gene *label is shown that this gene has no SD sequence (B), while in the NC_009512.Pput_2974 upstream gene label is shown that this gene has the SD sequence at 7 nucleotides to the start codon (C).

The second example is about the gene couple NC_002947.PP_2780 and NC_002947.PP_2781 of *Pseudomonas putida *KT2440 that overlap 130 bps. This overlap is the product of a misprediction of the start codon of the gene NC_002947.PP_2781 causing a 5'-end extension of the gene [35]. If we use the sequence of this gene as a query for the BLAST, we obtain as a first hit the orthologous gene NC_009512.Pput_2974, which is 127 bps shorter (compared with NC_002947.PP_2781) at the 5'-end and it is adjacent to the NC_009512.Pput_2975 gene (Figure 3). This gene pair (NC_009512.Pput_2974 and NC_009512.Pput_2975) belongs to *P. putida *F1 and overlaps only 4 bps, which is more reliable than the overlap of 130 bps. This is an example of a mispredicted overlap (NC_002947.PP_2780 and NC_002947.PP_2781) that could be corrected by just analyzing the BLAST results that we obtain automatically in this database, assuming that the orthologous gene has a correct prediction of the start site. In this case, the SD sequence prediction indicates that the NC_002947.PP_2781 gene has no SD sequence, while its orthologous gene (NC_009512.Pput_2974) has a strong SD sequence at 7 nucleotides to the start codon (Figure 3). Therefore, SD sequence location may help to expose wrong start codon predictions [13], as in the case of the NC_002947.PP_2781 gene, and to reinforce start codon predictions [38], as in the case of the orthologous gene (NC_009512.Pput_2974).

### Relationship between SD positions and the spacing lengths

**Figure 4:**
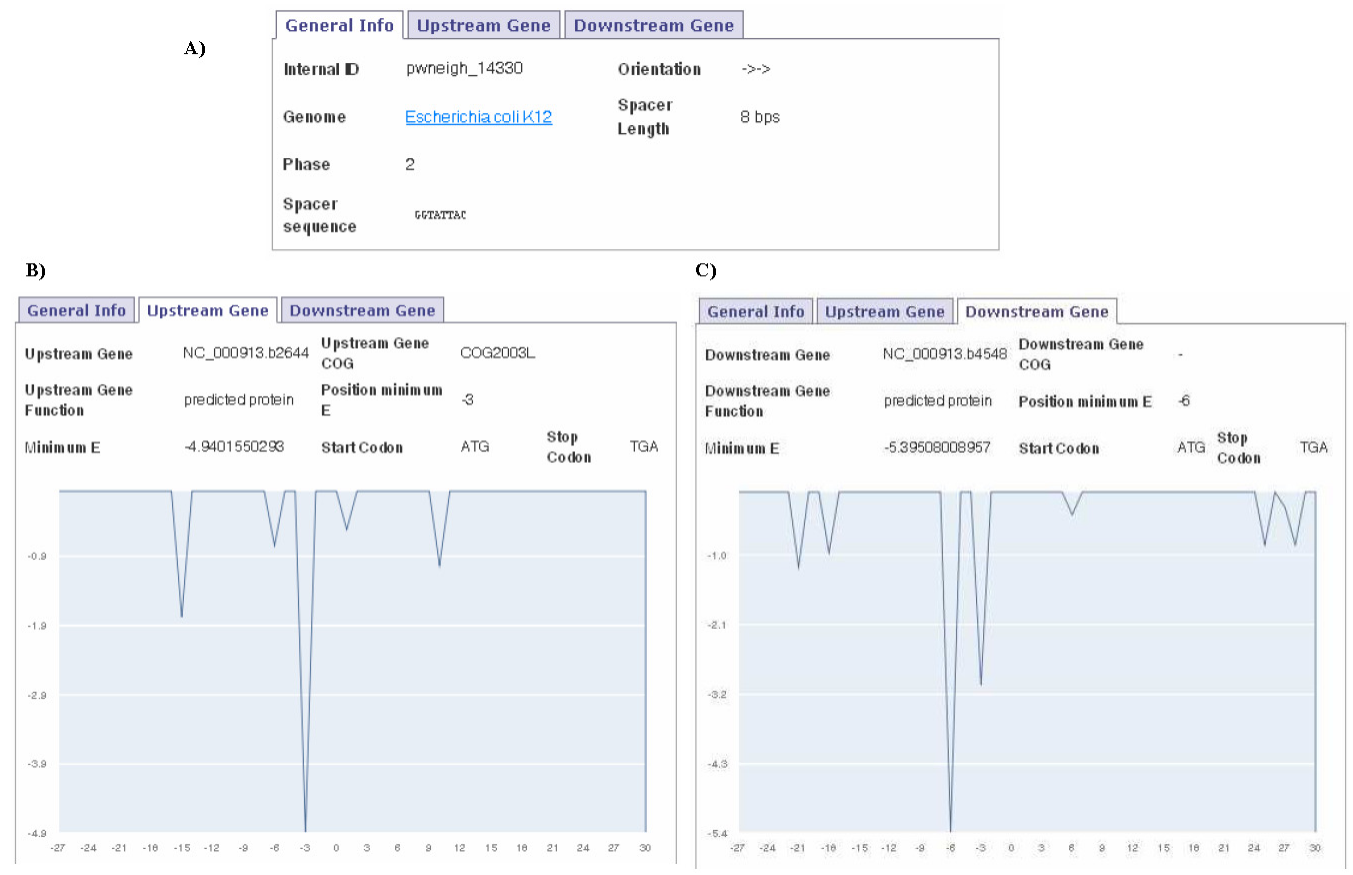
**Study of the location of the SD sequence between a co-directional gene pair**. Compilation of images that the users can find when they are studying the location of the SD sequence between the co-directional genes NC_000913.b2644 and NC_000913.b4548 separated by 8 bps. *General Info *label gives details about the spacer between this gene pair, which include the Spacing length and the Spacer sequence (A). The NC_000913.b2644 *Upstream Gene *label gives information about this gene (B), while the NC_000913.b4548 *Downstream Gene *label gives information about this gene as well as SD related information and the corresponding graph of the ΔG° values along the translation initiation region (C).

The third example is about the genes NC_000913.b2644 and NC_000913.b4548 of *E. coli *K12. These genes are separated by 8 bps (Figure 4), which is a short intergenic distance for a co-directional gene pair. The NC_000913.b4548 label shows that there is a drop in ΔG° value at 6 bps to the start codon (Figure 4). This means that the SD sequence of this gene is overlapping the upstream stop codon (TGA). If we join the upstream stop codon, the intergenic sequence and the downstream start codon we have the sequence TGAGGTATTACATG (Figure 4). The upstream stop codon is overlapping the SD motif resulting in the pattern TGAGGT that can bind with the SD sequence 3'-CCUCCA-5'. Therefore here we have detected a co-directional gene pair of *E. coli *K12 whose SD sequence for the downstream gene overlaps the upstream stop codon.

### SD presence among different gene sets

Other uses of the PairWise Neighbours database are to find out SD information of gene sets of interest, which have been labelled in other databases. For instance, the gene NC_000913.b3297 of *E. coli *K12 has been labelled as a *highly expressed gene *(HEG) in the HEG database [39]. This gene codifies for the 30S ribosomal protein S11 and has a strong SD sequence (the drop of ΔG° value is -11.44 Kcal/mol) at 10 nucleotides upstream to the start codon. If we analyse the SD presence in the *E. coli *K12 genes predicted as HEG in the HEG database (Table 1) [39], we find that the 81.03% of these genes have the SD sequence. This percentage is significantly higher compared with all the *E. coli *K12 genes (69.66%) and with the mean and standard deviation of the SD presence in 100 sets of 300 *E. coli *K12 genes randomly selected (69.04% ± 2.58%) (Table 1). Therefore, as other authors have already found [10], the HEGs appear to have more SD presence. Another interesting gene set that can be analysed in this database is the *horizontally transferred genes *(HGTs). We studied the SD presence among the *E. coli *K12 genes predicted as HGTs in the HGT database [40]. The percentage of HGTs that have SD sequence (68.39%) is close to the percentage of SD presence found in all the *E. coli *K12 genes. This percentage falls within the range of the mean and the standard deviation of 100 sets of 300 genes randomly selected from *E. coli *K12 (Table 1). Therefore, it seems that the HGTs have an equal SD presence to the original genes of the species.

**Table 1:** Genes with or without SD in *E. coli *K12

	Number of genes	Percentage of genes with SD	Percentage of genes without SD
All *E. coli *genes	4,133	69.66	30.34

*Highly expressed genes *(HEG) from *E. coli *^(1)^	253	81.03	18.97

*Horizontally transferred genes *(HGT) from *E. coli *^(2)^	310	68.39	31.61

Mean and standard deviation of 100 sets of 300 genes randomly selected from *E. coli*	300	69.04 ± 2.58	30.96 ± 2.58

Number of genes and percentage of genes with the Shine-Dalgarno motif from *E. coli *K12.

^(1) ^HEG extracted from the HEG-DB [39]

^(2) ^HGT extracted from the HGT-DB [40]

Abbreviations: SD, Shine-Dalgarno

## Conclusion

The studies of the translation initiation mechanism, gene regulation and gene structure (such operon predictions) rely on correct annotations. With the growing number of fully sequenced prokaryote genomes, the databases that help the annotation processes are very desirable. PairWise Neighbours is an interactive and intuitive database for retrieving information about the spacers and overlapping genes among bacterial and archaeal genomes. With this information, on the one hand, it is possible to study the reliability of an overlap as well as its conservation across the species with a BLAST local system, which permits the user to study the conservation of an overlap applying their desired Expect threshold. On the other hand, with the information related to the SD sequence and the ΔG° values along the translation initiation region, the users can analyse the intergenic regions widely. They can check the reliability of the initiation site prediction, the SD location and the SD strength or the relationship between SD location and the spacing lengths. In addition, it is possible to analyse the gene functions using the COG classes and the SD predictions.

## Availability and requirements

Project name: pwneigh

Project home page: 

Operating systems: Platform independent

Programming language: Python and SQL

Other requirements: Python 2.5, mySQL 5.0, Apache 2.0 and TurboGears 1.0.7

Licence: Content by Creative commons and source code by GNU GPL

Any restrictions to use by non-academicians: None

## Authors' contributions

AP performed the necessary Perl Scripts to obtain the raw data. TR built the MySQL database and designed the web application. AP, SGV and AR participated in the analysis and interpretation of the data. AP drafted the manuscripts and SGV and AR revised it critically. Finally, all the authors read and approved the version to be published.
